# The Phytochemical Constituents and Pharmacological Activities of *Annona atemoya*: A Systematic Review

**DOI:** 10.3390/ph13100269

**Published:** 2020-09-24

**Authors:** Bassam S. M. Al Kazman, Joanna E. Harnett, Jane R. Hanrahan

**Affiliations:** The School of Pharmacy, Faculty of Medicine and Health, The University of Sydney, Camperdown, NSW 2006, Australia; bassam.alkazman@sydney.edu.au (B.S.M.A.K.); joanna.harnett@sydney.edu.au (J.E.H.)

**Keywords:** *Annona atemoya*, custard apple, nutraceutical, phytochemistry, bioactivity, pharmacological activity

## Abstract

*Annona atemoya* also known as the custard apple is a hybrid between two Annonaceae species: Cherimoya (*Annona cherimola*) and the sugar apple (*Annona squamosa*). It is widely cultivated in tropical and subtropical continents including north and south America, Asia, Africa and Australia. Despite becoming an increasingly important commercial fruit plant due to its’ creamy succulent flesh, compared to other Annonaceae species relatively few studies have investigated the phytochemistry and bioactivities of *A. atemoya*. Studies that evaluated *A. atemoya* extracts and its constituents were searched through the databases Scopus, Pubmed and Embase from inception to June 2020. Constituents of *A. atemoya* include alkaloids, flavonoids, terpenes and acetogenins. The results indicate that the constituents of *A. atemoya* possess cytotoxic, anti-angiogenic, hypolipidemic, antioxidant, anti-inflammatory and neuroprotective activities. However, many of these studies are currently limited in quality and further phytochemical and pharmacological studies are required.

## 1. Introduction

*Annona atemoya* is a commercially important fruiting plant belonging to the Annonaceae family [1]. *A. atemoya*, is widely cultivated in tropical and subtropical continents including southern and northern America, Asia, Africa and Australia [2], Spain and Israel [3]. *A. atemoya* is a hybrid between two Annonaceae species: Cherimoya (*Annona cherimola*) and the sugar apple (*Annona squamosa*). Hybridization was conducted by P. J. Webster in Florida (USA) between 1907 and 1908 [2,3]. However, natural hybridization is also believed to have occurred between 1850 and 1930 in Australia and Palestine [3]. The cultivars vary between countries e.g., United States of America (Bradley, Page, Keller, Priestly, Stremer and Caves) [2]; Brazil (African Pride, Pink’s Mammoth, Thompson and Gefner) [4]; Australia (Island Gem, Nielsen, Hillary White, Maroochy Gold, Pink’s Mammoth and African Pride) [2,5]; Israel (Jennifer, Kabri, Malalai, African Pride and Gefner) [2] and Thailand (Golden Flesh and Pet Pakchong) [5].

*A. atemoya* grows to approximately 7.5–10 m, it is and short-bunked with low drooping branches [2,3]. The leaves of *A. atemoya* are alternate, leathery, deciduous and less hairy than those of the parent cheimola, averaging 15 cm in length [2]. The flowers are triangular, long stalked and yellow in colour, approximately 6 cm long and 4–5 cm in width. The fruit is fleshy, pale to bluish green, and variable shape from heart-shaped to round (2–2.5 kg), with a bumpy skin and its surface covered with prominent angular areoles that have some protuberances [2,3]. The fruits are also characterized by a pleasant flavour, the flesh is white and the pulp can be easily separable from the seeds which are smooth and brown to black in colour [1,3,5,6]. In Australia, this hybrid is commonly known as the “custard apple” and the increasingly popular fruit is available in retail food outlets [3,7].

Various *Annona* species such as *Annona muricata* have documented indications for use in traditional and complementary medicine practice [8]. As a relatively recent hybrid, traditional uses of *A. atemoya* are lacking, with some limited anecdotal evidence that the leaves of *A. atemoya* are purchased from growers for making teas. Several phytochemical constituents have been isolated from different parts of the *A. atemoya* plant and assessed for their biological potential in both *vivo* and *vitro* studies [9,10,11,12]. The leaves, fruits and seeds of *A. atemoya* are the most widely studied for their chemical and pharmacological properties. Although *A. atemoya* is a hybrid of *A. squamosa* and *A. cherimola* and both species have reported uses in traditional and folkloric medicine [10,13,14,15], to date only limited studies have investigated the phytochemistry and bioactivities of *A. atemoya*. The aim of this review was to systematically search the literature to identify studies reporting on the phytochemical constituents and bioactivity of *A. atemoya*.

## 2. Methodology

### 2.1. Search Strategy

A systematic search of the literature published from inception to 9 June 2020 was performed by using the three electronic databases Scopus, Pubmed and Embase using the search terms: “Annona atemoya” OR “custard apple” OR acetogenins OR bullatacin AND phytochemistry OR “phytochemical constituents” AND “pharmacological effects” OR “biological activities” OR anti-cancer OR anti-bacterial OR antioxidant OR anti-angiogenic AND toxicity OR toxicology.

### 2.2. Inclusion and Exclusion Criteria

Studies reporting on the pharmacological properties of isolated compounds or whole extracts of *A. atemoya,* and/or studies which examined the phytochemical constituents of any parts of the plant (aerial part, leave, fruit, seed, stem and root) were considered for inclusion in this review. Finally, regards language, both English and non-English language (one Portuguese paper) were included. Articles that discussed isolation or biological activity of acetogenins, synthetic acetogenins and bullatacin were not included unless they had been isolated from *A. atemoya*.

### 2.3. Data Extraction

Data extracted included information relating to the pharmacological and phytochemistry of *A. atemoya:* method of extraction, solvent of extraction, analytical techniques used for identifying pure compounds, structure of pure compounds, pharmacological activity of both crude extracts and pure compounds, animal tested, cell line tested, dosage range, route of administration, duration of treatment, in vivo or in vitro study, and the reported outcomes.

## 3. Results

### 3.1. Eligible Studies

**Figure 1:**
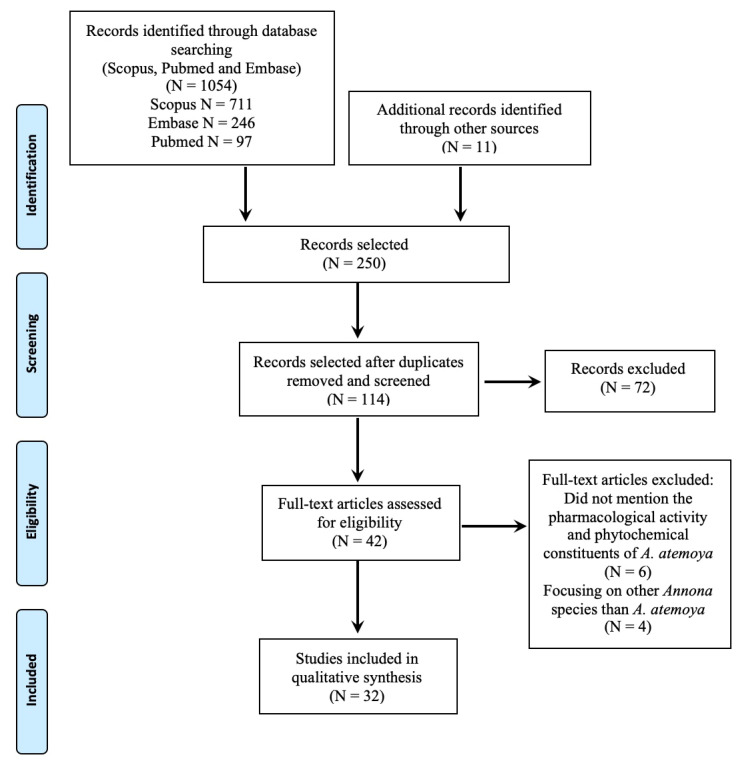
Preferred Reporting Items for Systematic Reviews aand Meta-Analyses (PRISMA) flowchart of literature search strategy and results.

As presented in Figure 1, a total of 1054 abstracts were identified in the initial search. After title and abstract screening and removal of duplicates, 114 studies were further evaluated for key terms and relevance to the aims of the review. Of the 114 abstracts identified, a further 72 articles were removed due to not meeting the inclusion criteria. A total 42 full text articles were evaluated resulting in a further 10 articles being removed due to not reporting pharmacological activity or phytochemical constituents or focusing on an *Annona* species other than *A. atemoya*. A total of 32 articles were included in this review. Of the 32 selected articles, 18 studies examined the phytochemical constituents of various parts of *A. atemoya* and 14 studies investigated the biological activities in vivo (*n* = *5*), in vitro (*n* = *7*) and two studies discussed its in vitro toxicity.

The quality assessment of animal studies was undertaken using the Animal Research: Reporting of In Vivo Experiments (ARRIVE) quality assessment criteria including ethical statement, study design, experimental procedure, experimental animals, housing and husbandry, simple size, allocating animals to experimental groups, experimental outcomes, statistical methods, baseline data, number of analysed, outcomes and estimation and adverse events [16]. Each ARRIVE quality assessment criteria was assessed as being met or not. Two authors agreed on the inclusion, exclusion criteria and search strategy. This systematic review was conducted by one author and reviewed by two authors.

### 3.2. Phytochemicals in A. atemoya

**Figure 2:**
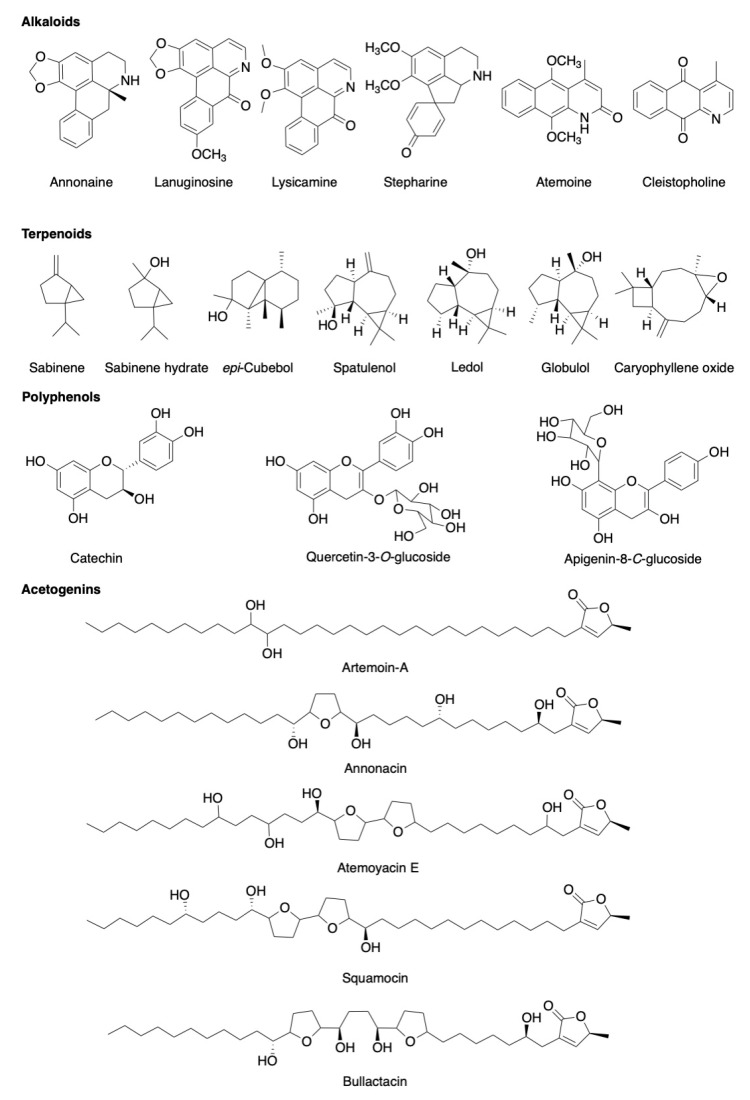
Structures of some representative phytochemical constituents of *Annona atemoya*.

As presented in Table S1, an extensive number of phytochemicals have been isolated from the parts of *A. atemoya*, the structures of some representative phytochemicals from *A. atemoya* are shown in Figure 2. The fruit has been reported to contain a wide range of volatile oils and phenolic compounds [17,18,19]. The seeds contain alkaloids and annonaceous acetogenins, and the leaves contain alkaloids and phenolic compounds [20,21,22,23,24,25,26]. *A. atemoya* is rich in the minerals iron, calcium, magnesium, zinc, and vitamins including thiamine, riboflavin, niacin, α-carotene, β-carotene, cryptoxanthin and ascorbic acid [19,26,27,28].

#### 3.2.1. Fruits

As the fruit of *A. atemoya* has a unique sweet and custard like aroma when ripe, the volatile constituents of *A. atemoya* have been investigated in many studies and the fruit is found to be the richer in volatiles compared to other parts of the plant [17,19]. Gas chromatography/mass spectrometry (GCMS) analysis of the essential oils from the mature, fresh and ripe fruits collected in South East Queensland Australia and stored at 25 °C for five days identified 40 phytochemical components [17]. They reported that 94% of the GC peak area contained esters, with methyl butanoate, ethyl butanoate and methyl hexanoate being the predominant constituents. The main terpenes from a total of 25 identified, were α-pinene, β-pinene, (*E*)-ocimene and germacrene D [17]. In a separate study, mono and sesquiterpene hydrocarbons were the main class of aromatic volatiles identified making up 77% and 80% of total volatile constituents identified [17]. The main compounds identified in these three studies were, α-pinene (70.4 ppm), limonene (44.2 ppm), β-pinene (27.3 ppm), germacrene D (20.0 ppm) and bornyl acetate (14.6 ppm) [6,7,17].

Additionally, another study reported the volatile constituents isolated from immature fruits of *A. atemoya* by various drying methods including solar drying and oven drying at 30 °C and at 50 °C [18]. The results of this study showed some similarities to the previously mentioned studies. Oven drying at 50 °C led to more volatile compounds being identified compared to the other methods. Both mono and sesquiterpene hydrocarbons were the largest group of phytochemicals identified [18].

Using liquid chromatography mass spectrometry/mass spectrometry (LC-MS/MS), the fruit of *A. atemoya* was shown to contain phenolic compounds including epicatechin and catechin at concentrations of 211 μg/g-dw and 38.6 μg/g-dw, respectively [27]. Other compounds were identified at minor concentrations: 3,4-dihydroxybenzoic acid (7.75 ± 0.18 μg/g-dw), chlorogenic acid (1.79 μg/g-dw) and *p*-coumaric acid (1.77 μg/g-dw. Caffeic acid and ferulic acid, rutin and quercetin were not detected in the samples tested in this study [27].

One study on the antitumour effects of bullatacin states that bullatacin is isolated from the fruit of *A. atemoya* [29], yet reference cited the paper by Chang et al. (1999) reporting bullatacin as being isolated from the seeds [20]. Given that acetogenins have not been reported as being isolated from *A. atemoya* fruit, it is most likely that the bullatacin was isolated from the seeds of the fruit.

#### 3.2.2. Leaves

The alkaloid constituents isolated by extraction with hexane and methanol from the leaves of *A. atemoya* collected in Petrolina in Brazil have been reported [30]. The phytochemical investigation of the methanol extract led to the identification of seven alkaloids: anonaine, asimilobine, lanuginosine, liriodenine, lysicamine, pronuciferine and stepharine were identified by mass spectrometry and nuclear magnetic resonance (NMR) spectroscopy. This was the first time that these alkaloids have been identified in the leaves of *A. atemoya* [30]. A recent study also reported similar compounds isolated from an 3:1 ethanol:water extract of the leaves of a commercial cultivar of *A. atemoya* grown in Sicily, however, the alkaloids anonaine and asimilobine, were not detected [31]. *A. atemoya* was also found to have comparable amounts of both oxoaporphine (liriodenine and lanuginoside) and proaporphine (stepharine and pronuciferine) alkaloids in the leaves [31]. In the same study, a number of phenolic compounds were isolated from the leaves of *A. atemoya* such as quercetin-3-*O*-rutinoside-7-*O*-glucoside, quercetin-3-*O*-rutinoside-7-*O*-pentoside, quercetin-3-*O*-rutinoside, kaempferol-3-galactoside-7-rhamnoside, quercetin-3-*O*-glucoside, kaempferol-3-*O*-glucoside, luteolin-3-galactoside-7-rhamnoside, luteolin-3-glucoside-7-rhamnoside, apigenin-8-*C*-glucoside, catechin and epicatechin [31].

#### 3.2.3. Seeds

There have also been limited studies on the phytochemical constituents from the seeds of the *A. atemoya*. According to many studies, the annonaceous acetogenins are the main phytochemical components of seeds compared to both phenolic and alkaloid constituents [20,21,22,23,24,25,32,33]. The seeds of *A. atemoya* collected in Taiwan, were crushed and extracted with EtOAc and then partitioned to yield aqueous and chloroform (CHCl_3_) extracts [20]. The CHCl_3_ layer was concentrated and partitioned between *n*-hexane and MeOH. A waxy residue (140 g) was collected from the MeOH extract and acetogenins were identified by testing with Kedde’s reagent [20]. The MeOH fraction was subjected to column chromatography and eluted with CHCl_3_, *n*-hexane and CHCl_3_ + MeOH resulted in 30 fractions. These fractions were re-chromatographed and then recrystallized using various solvent such as *n*-hexane: EtOAc (2:1) and MeOH:CHCl_3_ (20:1) [20]. The study identified 17 annonaceous acetogenins present in the seeds (12,15-*cis*-squamostatin-D, 12,15-*cis*-squamostatin-A, squamostatin-A, squamostatin-D, neoannonin, artemoin-A, artemoin-B, artemoin-C, artemoin-D, squamocin, bullatacin, bullatanocin, bullatalicin, 12,15-*cis*-bullatanocin, 12,15-*cis*-bullatalicin, desacetyluvaricin and isodesacetyluvaricin) and their structures were established [20].

A study of the seeds of *A. atemoya* collected in China and then extracted with 95% EtOH was carried out [32]. The residue was partitioned between H_2_O and EtOAc, to yield 12.5 g of a waxy compound from the organic phase. The wax was subsequently purified using a column chromatography with an eluent of cyclohexane and acetone (1:1) [32]. One fraction collected and then purified via preparative thin layer chromatography afforded a whitish wax (Atemoyacin A) [32].

Additionally, four studies examined the phytochemical constituents of seeds collected in Australia [21,22,23,24,25]. A total of 22 compounds were identified (annonisin, parviflorin, asimicin, cherimolin-1 and -2, mortrilin, molvizarin, rolliniastatin-1, annonacin, almunequin, atemoyin, desacetyluvaricin, squamocin, rolliniastatin-2, neoannonin, isodesacetyluvaricin, uvariamicin-III, annotemoyin-1 and 2, reticulatain-1, bulladecin and atemotetrolin) and their structures determined spectroscopically [21,22,23,24,25]. Finally, chromatographic purification of the ethanolic extracts of the seeds of *A. atemoya* afforded atemoyacin E [33].

As would be expected from a hybrid species, comparing the phytochemical constituents of *A. atemoya* with those identified and isolated from both *Annona cherimola* and *Annona squamosa,* indicate that all three species have some similarities in their constituents regardless of the parts of the plant from which they isolated from. For instant, various annonaceous acetogenins, alkaloids and volatile components have been isolated from *A. atemoya* and *A. squamosa* such as 12,15-*cis*-squamostatin-A, bullatacin, anonaine, α-Pinene, camphene, β-pinene, myrcene, spathulenol, germacrene D, squamostatin D, β-caryophyllene, uvariamicin-III, squamocin-G, squamocin-H, squamocin-J, squamocin-K, squamocin-L, squamocin-M; squamostatin-A, squamocin, annotemoyin-1, annotemoyin-2, liriodenine, annonacin, squamocin and molvizarin [34,35,36,37,38,39,40]. Anonaine, asimilobine, lysicamine, liriodenine, stepharine, α-pinene, camphene, β-pinene, myrcene, germacrene D, molvizarin, rolliniastatin-2, squamocin were also isolated from both *A. atemoya* and *A. cherimola* [13,41,42,43].

Both the fruits and seeds of *A. atemoya* contain a greater range and concentration of phytochemical constituents, and have been studied more extensively compared to the leaves. However, this should be interpreted within the context that there a fewer studies evaluating the *A. atemoya* leaves. The volatile constituents are the major components of the fruit, whereas the annonaceous acetogenins are the main components of the seed. Although the annonaceous acetogenins have been isolated from the seeds, no studies have reported the isolation of annonaceous acetogenins from the fruits or leaves. Alkaloids have been identified in both leaves and seeds, and phenolic components have been isolated from fruits and seeds. Therefore, it can be deduced from the studies included in this review, that the seed is the most diverse and richest source of phytochemicals including annonaceous acetogenins, alkaloids and phenolic compounds.

Despite the fact that many pure compounds have been isolated from *A. atemoya* and various extracts of different plant parts have been examined for their activity, knowledge about the pharmacological activities of *A. atemoya* remains relatively limited. Only fourteen studies included in this review evaluated the pharmacological activities of *A. atemoya*. Of the fourteen studies, only two studies reported the cytotoxic activities of pure compounds against various cancer cell lines [20,44], These compounds were isolated from the seeds and identified as annonaceous acetogenins which have been reported in other *Annona* species [20]. The remaining twelve studies described below, discuss the biological activity of crude extracts of different plant parts such as seed, leave, stem and fruit.

### 3.3. Pharmacological Properties of A. atemoya

Different extracts of *A. atemoya* and the phytochemical constituents isolated from several parts of the plant exhibit pharmacological activities such as anti-inflammatory and antinociceptive activity [45], antioxidant [11,31,46,47], antibacterial [46], anti-angiogenic [12], neuroprotective and anti-Alzheimer’s [9,11], anticancer [20,31,44], hypolipidemic [48] and antiobesity activity [49], in both in vivo and in vitro studies (Table 1).

#### 3.3.1. Cytotoxic Activity

Annonaceous acetogenins have attracted considerable attention due to their broad biological activities. Compounds isolated from the seeds of *A. atemoya* exhibit potent cytotoxicity against various cancer cell lines such as Hep G2/2.2.15, CCM2, CEM, KB and Hep G2 [20]. These compounds were identified as 12,15-*cis*-squamostatin-D, squamostatin-D, squamocin, neoannonin, bullatacin and desacetyluvaricin. Their activity on different cancer cells was assessed using a methylene blue colorimetric assay [20]. The cytotoxic activity of these compounds is presented in Table 2.

Bullatacin isolated from *A. atemoya* demonstrated a potent cytotoxic activity inhibiting the proliferation of Hep G2/2.2.15 cells by inducing apoptosis [29], via a reduction of intracellular cAMP and cGMP levels [44]. Using the methylene blue assay, a significant inhibition in Hep G2/2.2.15 cell proliferation was recorded after treatment with bullatacin (10^−1^ to 10^−5^ μM), ED_50_ values were recorded as 7.8 ± 2.5 nM for 24 h [44].

An aqueous-ethanolic extract of the leaves of *A. atemoya* was shown to be cytotoxic to both HeLa and HepG2 cancer cells. The anti-cancer activity was not found to correlate to either polyphenol content, antioxidant properties or total alkaloid content. However, there was a moderate correlation between antiproliferative activity and aporphine-type alkaloid content [31].

#### 3.3.2. Anti-Angiogenic Activity

The ethanolic extract of *A. atemoya* seeds was examined for its anti-angiogenic activity in human umbilical vein endothelial cells (HUVECs). The ethanolic seed extract of *A. atemoya* elicited a significant dose-dependent inhibition of HUVEC mobility at concentration ≥100 µg/mL without decreasing cell viability [12]. Using the matrigel plug assay, the ethanol extract at concentration ≥25 µg/mL was also shown to inhibit the formation of new blood vessels in vivo, in a dose-dependent manner. Further investigation indicated that under hypoxic conditions the extract down-regulated the expression of vascular endothelial growth factor (VEGF) and hypoxia-inducible factor (HIF-1alpha/2alpha) [12]. These results indicate that the extract modulated angiogenesis both directly through the endothelial cells and indirectly though modulating the tumour-induced angiogenic pathways [12].

#### 3.3.3. Hypolipidemic Effect

The ethanolic fruit extract of *A. atemoya* was investigated for its hypolipidemic effects using female KKAy mice at one month of age [48]. For four weeks, the female KKAy mice were fed a high fat diet. Oral administration of ethanolic fruit extract of *A. atemoya* at doses of 125 and 500 mg/kg to the female KKAy mice led to a significant inhibition of plasma triglyceride concentrations and the hypolipidemic effect might be through diminished fatty acid mobilization [48]. The hypolipidemic mechanism of the ethanolic fruit extract is not reported, although the authors claim that in a previous study [49], both the hexane and ethanolic extracts of *A. atemoya* fruit (along with many other popular Okinawa food stuffs) inhibited adipogenesis activity in 3T3-L1 cells by 50% or more. However, this study provides only limited results and does not include the extraction methodology or the concentrations of the extracts used, making it difficult to understand if this is a likely mechanism for the anti-obesity actions of *A. atemoya* [49]. While interesting, the dose of the crude extract used in the hypolipidemic study is high, translating into a dose of approximately 9 g/per day for an average adult. However, the significant issues faced by many western populations due to obesity and related health issues mean that the hypolipidemic activities warrant further study. In particular, further purification of the extract and isolation of the hypolipidemic constituent(s) is required.

#### 3.3.4. Antioxidant Activity

In an in vitro study, the methanolic seed extract of *A. atemoya* was examined for antioxidant potential [47]. Both 2,2-diphenyl-1-picrylhydrazyl (DPPH) free radical scavenging and oxygen radical absorbance capacity (ORAC) assays were employed. For the ORAC and DDPH assays, the Trolox equivalent antioxidant activity of the methanolic seed extract was recorded at 46.14 ± 1.25 and 4.82 ± 0.32 μmol TE g^−1^, respectively [47].

Other studies have reported the free radical scavenging activity of ethanolic extracts of stems and the hexane extract of leaves using DPPH, 2,2′-azino-bis(3-ethylbenzothiazoline-6-sulfonic acid (ABTS) and antioxidant activity β-carotene/linoleic acid assays [46]. The ethanolic extract of stems exhibited the most effective antioxidant in DPPH and ABTS (IC_50_ = 10.44 ± 1.25 µg/mL) and (24.81 ± 0.49%), respectively. The hexane leaf extract of *A*. *atemoya* has also demonstrated antioxidant activity in the β-carotene linoleic acid assay with approximately (41.12 ± 4.35%) [46]. The aqueous ethanol leaf extracts of Sicilian *A. atemoya* also showed strong antioxidant activity in the ABTS (5.01 TE g^−1^), DPPH (13.51 TE g^−1^) and metal reducing properties in the ferric reducing antioxidant power (FRAP) assay (14.79 TE g^−1^) [31].

#### 3.3.5. Antibacterial Activity

The antimicrobial potential of the hexane and methanolic extract of leaves, and the ethanolic extract of the stems of *A*. *atemoya* has been investigated against both Gram-positive and Gram-negative bacteria using a serial dilution protocol [46]. All strains were resistant to the hexane extract. However, both the leaf methanolic and stem ethanolic extracts exhibited activity against gram positive *Staphylococcus epidermidis, Bacillus cereus, Staphylococcus aureus* and a clinical isolate of *methicillin-resistant Staphylococcus aureus* (MRSA), with *Klebsiella pneumoniae* the only Gram-negative strain sensitive to the extract. The minimum bactericidal concentration (MBC) values recorded for the ethanolic stem extract ranged from 781 µg/mL against MRSA to 6250 µg/mL against *K. pneumoniae* and *S. aureus*. The methanolic leaf extract showed weaker activity with MBC values ranging from 3125 against *S. epidermidis* to 12,500 µg/mL (*S. aureus*, *K. pneumoniae* and *B. cereus*) [46]. Although, the MBC was determined, the minimum inhibitory concentration (MIC) was not determined for any of the strains. Therefore, it is not possible to determine whether the mechanism of action is bactericidal or bacteriostatic. From the reported total polyphenol content of these extracts it is suggest that flavonoids are responsible for the activity antibacterial action of both the ethanol stem extract and the methanol leaf extract [46].

#### 3.3.6. Antinociceptive Activities

An in vivo study evaluating the antinociceptive activity of *A. atemoya* was conducted using the acetic acid-induced writhing and formalin mouse models [45]. The ethanolic extract of leaves (25, 50 and 100 mg/kg) inhibited acetic acid-induced writhing by 42.14, 48.88 and 63.48%, correspondingly. The same doses of the ethanolic extract of leaves also exhibited antinociceptive activity with 56.71, 64.35 and 50.09% inhibition, respectively using the formalin test [45].

#### 3.3.7. Anti-Inflammatory Activities

The anti-inflammatory activity of *A. atemoya* was reported in a single study with the evaluation carried out using the air pouch and carrageenan-induced peritonitis models in an in vivo study [45]. In the carrageenan-induced peritonitis model the ethanolic extract of leaves (25, 50 and 100 mg/kg) inhibited leukocyte migration by 35.40, 46.20 and 63.85%, respectively. In the air pouch assay, the leaf extract (25, 50 and 100 mg/kg) also reduced leukocyte migration into the air pouch by 56.17, 62.04 and 73.16% respectively [45]. As this study used only the crude the ethanolic extract it is not possible to identify which constituents may be responsible for the antinociceptive and anti-inflammatory activities.

#### 3.3.8. Neurological Activities

Oral administration of an ethanolic extract of *A. atemoya* was found to reduce scopolamine- induced memory impairment in the mouse Y-maze and passive avoidance tests [9,11]. Investigation into the mechanism of action indicated that *A. atemoya* extract inhibits memory impairment by preventing cholinergic dysfunction and decreasing cell death associated with stress induced oxidation [9]. The crude ethanolic leaf extract and to a lesser degree the fruit extract but not the seed extract was also shown to prevent amyloid-β (Aβ) aggregation and increase free radical scavenging activity, preventing H_2_O_2_ induced damage to hippocampal HT22 cells in vitro [9]. Immunohistochemical and western blotting studies indicated that the extract inhibited Aβ-mediated loss of brain derived neurotrophic factor (BDNF) expression and decreased Aβ-mediated phosphorylation of epidermal growth factor receptor (EGFR) and G Protein-Coupled Receptor Kinase 2 (GRK2) [9]. Further fractionation into hexane, butanol and water led to all fractions inhibiting Aβ aggregation to some degree, with the ethyl acetate fraction demonstrating the greatest inhibition. However, the majority of the neuroprotective activity was found in the butanol fraction with the hexane and ethyl acetate fractions (the fractions most likely to contain the lipophilic acetogenins) being cytotoxic to the cultured neurons. Phytochemical analysis of the butanol extract suggested that the flavonoids rutin and isoquercitrin were predominantly responsible for the reported bioactivity [9].

However, one study has reported the neurotoxicity of ethyl acetate extracts of the pulp and seeds in an in vitro study using Lund human mesencephalic (LUHMES) cells [50]. This suggests that constituents of the seeds and pulp are neurotoxic as has been found for other members of the *Annona* family [2]. There have been suggestions that a high dietary consumption of *A. muricata* may be linked a Parkinson disease-like syndrome possibly due to the annonacin content [52]. The studies investigating the neurotoxicity of *Annona* species have used ethyl acetate extracts which would predominantly contain the acetogenins, or purified acetogenins such as annonacin. Acetogenins have been identified as highly potent inhibitors of mitochondrial complex I [53], and this has been suggested as the mechanism of neurotoxicity. The neuroprotective effect of crude ethanolic extracts of leaves and fruit of *A. atemoya*, containing both acetogenins and flavonoids, suggests that the presence of the flavonoids may offer protection from the neurotoxic effects of the acetogenins.

#### 3.3.9. Toxicity

The neurotoxicity of the ethyl acetate extracts of a range of Brazilian *A. atemoya* pulp and seeds in LUHMES cells was assessed using both MTT assay to determine cell viability and LDH assay to determine cell death [50]. The pulp extract of *A. atemoya* had little effect on cell viability of the LUHMES cells at 1µg/mL, but at 10 µg/mL decreased cell viability to 12.7% ± 3.7%. The seed extract of *A. atemoya* exhibited significantly higher toxicity with cell viability reduced to 4.0% ± 0.8% at a concentration of 0.1 µg/mL. LDH levels were increased to 52.8% ± 3.9% by treatment of the LUHMES cells with concentration of 10 µg/mL of the *A. atemoya* pulp extract and to 60.8% ± 4.3% when treated with 0.1 µg/mL of seed extract [50]. When compared to the fruit pulp extract of other *Annona* species in the study *A. atemoya* had a lesser effect on cell viability than *A. squamosa* but resulted in a similar increase in LDH indicating a similar effect on cell death. This is despite *A. atemoya* containing a similar amount of annonacin to *A. squamosa* (3.8 of 2.2 µg/g dry weight) and approximately a 100-fold greater amount of squamocin [50]. The ethyl acetate extract used in this study will contain a complex mixture of lipophilic compounds, including many acetogenins, and the neurotoxicity cannot be attributed only to annonacin. It is possible that *A. atemoya* may also contain lipophilic neuroprotective compounds.

A second study reported the toxicity of *A. atemoya* on *Trichoplusia ni* (cabbage looper larva) using topical and oral administration. The methanolic seed extract was applied to *T. ni* larvae and the LC_50_ was recorded at 197.7 µg/larva [51]. Finally, the methanolic seed extract also showed toxicity to *T. ni* larvae when administered orally with the LC_50_ recorded as 382.4 ppm [51].

### 3.4. Quality Analysis

As presented in Table 3, the quality of the in vivo studies none of the studies met all of the ARRIVE criteria [16]. In particular, no studies reported baseline animal data or adverse events. With regards allocating animals to experimental groups, this was reported by only one group [9]. These factors limit the reliability of our findings in terms of the in vivo studies.

## 4. Conclusions

To our knowledge, this is the first systematic review to summarize the available literature reporting the phytochemical constituents and pharmacological effects of *A. atemoya.* The systematic review of the included studies affords a comprehensive report of the currently available data regarding both the phytochemical constituents and biological activities of *A. atemoya.* The selected studies in this systematic review were judged to be of medium quality as there are limited animal studies and no human studies reported. The phytochemical components of *A. atemoya* have been examined as extracts and pure compounds in both in vivo and in vitro. However, many studies used only crude ethanolic or methanolic extracts and did not attempt to purify these further in order to identify which constituents may be responsible the activities. In regard to the phytochemical screening of *A. atemoya* plant, several studies have reported a range of constituents isolated from various parts including the leaves, fruits and seeds. However, very few of the studies attempted to profile or quantify the bioactive constituents. Amongst the selected studies, only three studies reported the cultivar of *A. atemoya* (African Pride) one of the most common varieties [19,21,22]. It is also of interest to note that at the current time no acetogenins have been isolated from the fruit or leaves of *A. atemoya*, unlike the many acetogenins isolated from the fruit and leaves of *A. cherimola* and *A. squamosa*.

Although, there is no solid evidence medicinal or nutraceutical uses of *A. atemoya* there is some limited anecdotal evidence reporting the use of leaves for making teas in a traditional manner, or possibly using the leaves as a supplement. As an increasingly important commercial fruit crop with nutraceutical potential, further studies are recommended in order to isolate and investigate the pure components and identify the bioactive compounds responsible for the effects identified.

## Figures and Tables

**Table 1:** Plant part, extraction methods and biological activities and results.

Plant Part	Extraction Method/Type	Bioactivity	Model	Main Results	Ref.
Leaves	Ethanolic extract	Neuroprotective	In vitro: anti-oxidant activity	Dose-dependently (6.25–100 mg/mL) enhanced scavenging activity against ABTS and DPPH radicals	[9]
		Neuroprotective	In vitro: HT22 neuronal cell death	Extract significantly reversed H_2_O_2_-induced neuronal cell death at 25 or 50 µg/mL	[9]
		Neuroprotective	In vivo: Aβ-injected AD like mouse-model	Increased expression of NeuN and BDNF in hippocampus reversing the effects of intracerebroventricular injection of Aβ aggregates	[9]
		Neuroprotective	In vivo: Aβ-injected AD like mouse-model	Reduced the Aβ-mediated phosphorylation of EGFR and GRK2	[9]
		Anti-Alzheimer’s	In vitro: Aβ aggregation	Dose-dependently inhibited Aβ aggregation by 91.35% at 100 mg/mL	[9]
		Anti-Alzheimer’s	In vivo: Aβ-injected AD like mouse-model	At 100 μg/mL extract significantly attenuated the effects of Aβ aggregation in the passive avoidance task and Y-maze test	[9]
		Neuroprotective	In vivo: SCO-induced hippocampal neuronal damage	Prevented scopolamine-induced neuron damage in SCO-mediated memory deficit mice as shown by cresyl violet staining	[11]
		Neuroprotective	In vivo: cholinergic function in scopolamine-treated Mice	Increased acetylcholine content, choline acetyltransferase, and acetylcholinesterase activity in the hippocampus of SCO-treated mice	[11]
		Neuroprotective	In vivo: oxidative Stress in scopolamine-treated Mice	Attenuated the SCO-induced increase in reactive oxygen species (ROS) levels in the hippocampus	[11]
		Neuroprotective	In vivo: neuronal apoptosis in SCO-treated mice	Significantly decreased apoptotic activation in hippocampus of SCO-treated mice	[11]
		Anti-Alzheimer’s	In vivo: SCO-induced cognitive deficit mouse model	Significantly attenuated the memory deficits from scopolamine treatment in passive avoidance task and Y-maze test	[11]
		Antioxidant	In vitro: ABTS and DPPH free radical scavenging assays	At 100 μg/mL, AALE dose-dependently enhanced scavenging activity against ABTS and DPPH radicals by 97% and 82% respectively.	[11]
		Antioxidant	In vitro: ABTS, DPPH and FRAP free radical scavenging assays	ABTS 5.01 TE g^−1^ DPPH 13.51 TE g^−1^ 14.79 TE g^−1^	[31]
		Anticancer	In vitro: cytotoxicity HeLa, HepG2 cells	GI_50_ ~ 2 µg/mL	[31]
		Antinociceptive activity	In vivo: acetic acid-induced writhing and formalin mouse models	AAIW 100 mg/kg inhibited writhing 63.48% FMM 100 mg/kg inhibited pain response 63.48%	[45]
		Anti-inflammatory	In vivo: air pouch mouse model In vivo: carrageenan-induced peritonitis mouse models	100 mg/kg inhibited leukocyte migration in to air-pouch by 73.16% 100 mg/kg inhibited leukocyte migration by 63.85%	[45]
	Methanolic extract	Antibacterial	*In vitro*: against strains of *S. epidermidis, B. cereus*, methicillin-resistant *S. aureus*, *K. pneumoniae and S. aureus*.	MBC range 3125 to 12,500 µg/mL.	[46]
	Hexane extract	Antioxidant	*In vitro*: inhibition of β-carotene-linoleic acid bleaching assay	41.12 ± 4.35% inhibition	[46]
Seeds	Ethanolic extract	Anti-angiogenic	In vitro and in vivo models, involving cell proliferation, HUVEC and tumour-induced angiogenesis.	EEAA dose-dependently inhibited HUVEC proliferation at conc. ≥ 100 μg/mL.	[12]
		Anticancer	In vitro: cytotoxicity Hep G_2_, Hep 2,2,15, KB, CCM_2_ and CEM cells	Isolated acetogenins ED_50_ from 2.2 × 10^−4^ to > 500 µg/mL	[20,44]
		Neurotoxicity	In vitro: LUHMES cells	0.1 µg/mL reduced cell viability to 4.0% ± 0.8%	[50]
	Methanolic	Antioxidant	ABTS and DPPH free radical scavenging assays	46.14 ± 1.25 and 4.82 ± 0.32 μmol TE g^−1^	[47]
		Larvicidal	In vitro: *Trichoplusia ni*	Topical LC_50_ 197.7 µg/larva Oral LC_50_ 382.4 ppm	[51]
Stem	Ethanolic extract	Antioxidant	In vitro: ABTS and DPPH free radical scavenging assays	DPPH; IC_50_ = 10.44 ± 1.25 µg/mL ABTS; IC_50_ = 24.81 ± 0.49%	[46]
		Antibacterial	In vitro: against *S. epidermidis, B. cereus, methicillin-resistant S. aureus*, *K. pneumoniae, S. aureus.*	MBC range 781–6250 µg/mL.	[46]
Fruits	Ethanolic extract	Hypolipidemic Effect	In vivo: oral administration of extracts to Female KKAy mice (5 weeks of age) fed a high fat diet for 4 weeks	Significantly lowered the plasma triglyceride (TG) concentration at doses of 125 and 500 mg/kg.	[48]
	Ethanolic and hexane extracts	Anti-Obesity Activity	In vitro: 3T3-L1 cell line	50% or more inhibition of adipogenesis in 3T3-L1 cells.	[49]
	Ethanolic extract	Neurotoxicity	In vitro: LUHMES cells	10 µg/mL decreased cell viability to 12.7% ± 3.7%	[50]

AAIW acetic acid induced writing; Aβ β-amyloid; ABTS 2,2′-azino-bis(3-ethylbenzothiazoline-6-sulfonic acid; AD Alzheimer’s disease; AALE *A. atemoya* leaf extract; BDNF brain derived neurotropic factor; DPPH 2,2-diphenyl-1-picrylhydrazyl; EEAA ethanol extract *A. atemoya*; EGFR epidermal growth factor receptor; FMM formalin mouse model; FRAP ferric reducing antioxidant power; GRK2 G protein-coupled receptor kinase 2; HUVEC: human umbilical vascular endothelial cells; MBC minimum bactericidal concentration; NeuN neuronal nuclear protein; ROS reactive oxygen species; SCO scopolamine.

**Table 2:** Isolated pure compounds from *A. atemoya* and their cytotoxic activities [20].

Compound	(ED_50_ µg/mL)				
	Hep G2	Hep 2,2,15	KB	CCM2	CEM
12,15-*cis*-Squamostatin-D	2.20 × 10^−4^	3.10 × 10^−3^	4.05 × 10^−4^	-	-
Squamostatin-D	1.50 × 10^−4^	1.50 × 10^−3^	3.90 × 10^−4^	-	-
Squamocin	8.80 × 10^−4^	1.50 × 10^−3^	2.70 × 10^−1^	1.60 × 10^−2^	149
Neoannonin	1.10 × 10^−4^	1.26 × 10^−4^	1.46 × 10^−4^	10.9	520
Bullatacin	9.70 × 10^−5^	1.11 × 10^−4^	1.17 × 10^−4^	1.41 × 10^−1^	169
Desacetyluvaricin	1.02 × 10^−4^	1.18 × 10^−4^	1.35 × 10^−4^	23.5	100

**Table 3:** Methodological quality assessment of animal studies using ARRIVE quality assessment criteria.

Study	* Arrive Quality Items												
	1	2	3	4	5	6	7	8	9	10	11	12	13
[9]	Y	Y	Y	Y	Y	Y	Y	Y	Y	N	N	Y	N
[11]	Y	Y	Y	Y	Y	Y	N	Y	Y	Y	N	Y	N
[12]	Y	Y	Y	Y	N	N	N	Y	N	N	N	Y	N
[48]	N	Y	Y	Y	Y	N	N	Y	N	N	N	Y	N
[45]	Y	Y	Y	Y	Y	Y	N	Y	Y	Y	N	Y	N

* Arrive quality items: 1 Ethical statement, 2 Study design, 3 Experimental procedure, 4 Experimental animals, 5 Housing and husbandry, 6 Sample size, 7 Allocating animals to experimental groups, 8 Experimental outcomes, 9 Statistical methods, 10 Number of analysed, 11 Baseline data, 12 Outcomes and estimation, 13 Adverse events.

## Supplementary Materials

The following are available online at https://www.mdpi.com/1424-8247/13/10/269/s1, Table S1: Phytochemical constituents found in different parts of the *Annona atemoya* plant.
