# Supplementary material for: The Phytochemical Constituents and Pharmacological Activities of Annona atemoya: A Systematic Review
Source: Pharmaceuticals (Basel). 2020 Sep 24;13(10):269. doi: 10.3390/ph13100269 (PMC7598603; doi:10.3390/ph13100269)
Supplement: Supplementary file 1 [file pharmaceuticals-13-00269-s001.pdf]

The phytochemical constituents and pharmacological activities of *Annona atemoya*: A systematic review

Bassam S.M. Al Kazman[a], Joanna E. Harnett[a], Jane R. Hanrahan[a,*]

[a]The School of Pharmacy, Faculty of Medicine and Health, The University of Sydney, Camperdown, NSW 2006, Australia.

Supporting Information

Table S1. Phytochemical constituents found in different parts of the *Annona atemoya* plant.

Plant part	Chemical class	Phytochemical constituents	Reference
Leaf	Alkaloids	Anonaine<br>Asimilobine<br>Lanuginosine<br>Liriodenine<br>Lysicamine<br>Proaporphine<br>Pronuciferine<br>Stepharine	[1, 2]
	Flavonoids	Quercetin-3-*O*-rutinoside-7-*O*-glucoside<br>Quercetin-3-*O*-rutinoside-7-*O*-pentoside<br>Quercetin-3-*O*-rutinoside,<br>Kaempferol-3-galactoside-7-rhamnoside<br>Quercetin-3-*O*-glucoside<br>Kaempferol-3-*O*-glucoside<br>Luteolin-3-galactoside-7-rhamnoside<br>Luteolin-3-glucoside-7-rhamnoside<br>Apigenin-8-*C*-glucoside<br>Catechin<br>Epicatechin	[1]
Fruit	Terpenes	α-Pinene<br>β-Pinene<br>Camphene<br>Myrcene<br>α-Phellandrene<br>α-Terpinene<br>Limonene<br>β-Phellandrene<br>1,8-Cineole<br>(*Z*)-Ocimene<br>β-Elemene<br>Terpinen-4-ol<br>β-Caryophyllene<br>Aromadendrene<br>(Z)-p-menth-2-en-l-ol<br>(*E*)-p-Menth-2-en-l-o1<br>Isopinocarveol<br>α-Humulene<br>α-Terpineol<br>Borneol<br>Viridiflorene<br>(*E*)-Ocimene<br>p-Cymene<br>Terpinolene<br>α-Dimethylstyrene<br>β-Dimethylstyrene<br>α-Cubebene<br>β-Cubebene<br>α-Copaene	[3-7]

		Linalool	
		Bornyl acetate	
		Germacrene D	
		Bicyclogermacrene	
		δ-Cadinene	
		Globulol	
		Viridiflorol	
		Spathulenol	
		epi-Muurolol	
		δ-Cadinol	
		α-Cadino1	
		epi-Cadinol	
		Sabinene	
		Sabinene hydrate	
		α-Terpinolene	
		Myrtenal	
		cis-Verbenol	
		trans-Verbenol	
		trans-Pinocarveol	
		β-Selinene	
		γ-Cadinene	
		Myrtenol	
		trans-Carveol	
		Calamenene	
		epi-Cubebol	
		Caryophyllene oxide	
		Ledol	
		α-Kaurene	
	Phenolic compounds	3,4-Dihydroxybenzoic acid	[4]
		p-Coumaric acid.	
		Chlorogenic acid	
		Catechin	
		Epicatechin	
Seeds	Acetogenins	Almunequin	[8-15]
		Annonacin	
		Annonisin	
		Annotemoyin-1	
		Annotemoyin-2	
		Asimicin	
		Artemoin-A	
		Artemoin-B	
		Artemoin-C	
		Artemoin-D	
		Atemotetrolin	
		Atemoyacin-A	
		Atemoyacin-E	
		Atemoyin	
		Bulladecin	
		Bullatacin	
		Bullatalicin	
		12,15-cis-Bullatalicin	

		Bullatanocin<br>12,15-*cis*-Bullatanocin<br>Cherimolin-1<br>Cherimolin-2<br>Desacetyluvaricin<br>Isodesacetyluvaricin<br>Motrilin<br>Molvizarin<br>Neoannonin<br>Parviflorin<br>Reticulatain-l<br>Rolliniastatin-2<br>Squamocin<br>Squamostatin A<br>12,15-*cis*-Squamostatin A<br>squamostatin D<br>12,15-*cis*-Squamostatin D<br>Uvariamicin-III	
	Phenolic compounds	Chlorogenic acid<br>Ferulic acid<br>Vanillic acid<br>Myricetin	[16]
	Alkaloids	Atemoine<br>Cleistopholine.	[17]
	N-acyl tryptamines	*N*-Nonadecanoyltryptamine<br>*N*-Behenoyltryptamine<br>*N*-Lignoceroyltryptamine<br>*N*-Cerotoyltryptamine<br>*N*-Octacosanoyltryptamine<br>*N*-Tricosanoyl-4,5-dihydroxytryptamine<br>*N*-Lignoceroyl-4,5-dihydroxytryptamine<br>*N*-Pentacosanoyl-4,5-dihydroxytryptamine<br>*N*-Heptacosanoyl-4,5-dihydroxytryptamine	[17]
Roots	Acetogenins	Atemoyacin C	[18]

References

1. Mannino, G.; Gentile, C.; Porcu, A.; Agliassa, C.; Caradonna, F.; Bertea, C. M., Chemical Profile and Biological Activity of Cherimoya (*Annona cherimola* Mill.) and Atemoya (*Annona atemoya*) Leaves. *Molecules* **2020,** 25, (11), 2612.
2. Rabêlo, S. V.; Costa, E. V.; Barison, A.; Dutra, L. M.; Nunes, X. P.; Tomaz, J. C.; Oliveira, G. G.; Lopes, N. P.; Santos, M. d. F. C.; Almeida, J. R., Alkaloids isolated from the leaves of atemoya (*Annona cherimola* × *Annona squamosa*). *Rev. Brasi. Farmacogn.* **2015,** 25, (4), 419-421.
3. Bartley, J., Volatile constituents of custard apple. *Chromatographia* **1987,** 23, (2), 129-131.

4. de Moraes, M. R.; Ryan, S. M.; Godoy, H. T.; Thomas, A. L.; Maia, J. G. S.; Richards, K. M.; Tran, K.; Smith, R. E., Phenolic Compounds and Metals in Some Edible Annonaceae Fruits. *Biol. Trace Elem. Res.* **2020**, 1-7.
5. Liu, T.-T.; Chao, L. K.-P.; Peng, C.-W.; Yang, T.-S., Effects of processing methods on composition and functionality of volatile components isolated from immature fruits of atemoya. *Food Chem.* **2016,** 202, 176-183.
6. Pino, J. A.; Rosado, A., Volatile constituents of custard apple (*Annona atemoya*). *J. Essent. Oil Res.* **1999,** 11, (3), 303-305.
7. Wyllie, S. G.; Cook, D.; Brophy, J. J.; Richter, K. M., Volatile flavor components of *Annona atemoya* (custard apple). *J. Agric. Food Chem.* **1987,** 35, (5), 768-770.
8. Chang, F.-R.; Chen, J.-L.; Lin, C.-Y.; Chiu, H.-F.; Wu, M.-J.; Wu, Y.-C., Bioactive acetogenins from the seeds of *Annona atemoya*. *Phytochemistry* **1999,** 51, (7), 883-889.
9. Chiu, H.-F.; Chih, T.-T.; Hsian, Y.-M.; Tseng, C.-H.; Wu, M.-J.; Wu, Y.-C., Bullatacin, a potent antitumor Annonaceous acetogenin, induces apoptosis through a reduction of intracellular cAMP and cGMP levels in human hepatoma 2.2.15 cells. *Biochem. Pharmacol.* **2003,** 65, (3), 319-327.
10. Duret, P.; Hocquemiller, R.; Cavé, A., Annonisin, a bis-tetrahydrofuran acetogenin from *Annona atemoya* seeds. *Phytochemistry* **1997,** 45, (7), 1423-1426.
11. Duret, P.; Hocquemiller, R.; Cavé, A., Bulladecin and atemotetrolin, two bis-tetrahydrofuran acetogenins from *Annona atemoya* seeds. *Phytochemistry* **1998,** 48, (3), 499-506.
12. Duret, P.; Hocquemiller, R.; Laurens, A.; Cave, A., Atemoyin, a new bis-tetrahydrofuran acetogenin from the seeds of *Annona atemoya*. *Nat. Prod. Lett.* **1995,** 5, (4), 295-302.
13. Duret, P.; Waechter, A.-I.; Hocquemiller, R.; Cave, A.; Batten, D., Annotemoyin-1 and-2: two novel monotetrahydrofuranic γ-lactone acetogenins from the seeds of *Annona atemoya*. *Nat. Prod. Lett.* **1996,** 8, (2), 89-95.
14. Duret, P.; Waechter, A.-I.; Margraff, R.; Foucault, A.; Hocquemiller, R.; Cavé, A., High-Speed Countercurrent Chromatography: A Promising Method for the Separation of the Annonaceous Acetogenins. *J. Liq. Chromatogr. Relat. Technol.* **1997,** 20, (4), 627-635.
15. Wu, P.; Chen, W.-S.; Hu, T.-S.; Yao, Z.-J.; Wu, Y.-L., Atemoyacin E, a bis-tetrahydrofuran annonaceous acetogenin from *Annona atemoya* seeds. *J. Asian Nat. Prod. Res.* **2001,** 3, (3), 177-182.
16. Vagula, J. M.; Visentainer, J. V.; Lopes, A. P.; Maistrovicz, F. C.; Rotta, E. M.; Suzuki, R. M., Antioxidant activity of fifteen seeds from fruit processing residues by different methods. *Acta Scientiarum. Technology* **2019,** 41, e35043.
17. Wu, Y.-C.; Chang, F.-R.; Chen, C.-Y., Tryptamine-Derived Amides and Alkaloids from the Seeds of *Annona atemoya*. *J. Nat. Prod.* **2005,** 68, (3), 406-408.
18. Wu, P.; Chen, W.; Yu, Q.; Wu, Y., Annonaceous acetogenins from roots of *Annona atemoya* Hort. *Chin. J. Org. Chem.* **1999,** 19, (1), 46-52.
